# A novel technique to measure chronic levels of corticosterone in turtles living around a major roadway

**DOI:** 10.1093/conphys/cou036

**Published:** 2014-08-16

**Authors:** James H. Baxter-Gilbert, Julia L. Riley, Gabriela F. Mastromonaco, Jacqueline D. Litzgus, David Lesbarrères

**Affiliations:** 1 Department of Biology, Laurentian University, 935 Ramsey Lake Road, Sudbury, ON, Canada P3E 2C6; 2 Reproductive Physiology, Toronto Zoo, 361A Old Finch Ave., Toronto, ON, Canada M1B 5K7

**Keywords:** Corticosterone, highway, reptile, road ecology, stress, turtle

## Abstract

Conservation biology integrates multiple disciplines to expand the ability to identify threats to populations and develop mitigation for these threats. Road ecology is a branch of conservation biology that examines interactions between wildlife and roadways. Although the direct threats of road mortality and habitat fragmentation posed by roads have received much attention, a clear understanding of the indirect physiological effects of roads on wildlife is lacking. Chronic physiological stress can lower immune function, affect reproductive rates and reduce life expectancy; thus, it has the potential to induce long-lasting effects on populations. Reptiles are globally in decline, and roads are known to have negative effects on reptile populations; however, it is unknown whether individual responses to roads and traffic result in chronic stress that creates an additional threat to population viability. We successfully extracted reliable measures of corticosterone (CORT), a known, commonly used biomarker for physiological stress, from claw trimmings from painted turtles (*Chrysemys picta*) captured at three study sites (road-impacted site, control site and validation site). Corticosterone levels in claws were evaluated as a measure of chronic stress in turtles because CORT is deposited during growth of the claw and could provide an opportunity to examine past long-term stress levels. While male turtles had higher CORT levels on average than females, there was no difference in the level of CORT between the road-impacted and control site, nor was there a relationship between CORT and turtle body condition. In validating a novel approach for non-invasive measurement of long-term CORT levels in a keratinized tissue in wild reptiles, our study provides a new avenue for research in the field of stress physiology.

## Abstract (precis)

Reptiles are globally endangered, and roadways are a major threat to many species. We extracted corticosterone from turtle claws to examine whether proximity to roads increased stress levels. Our novel sampling method was successful; however we found no difference in corticosterone levels between road-adjacent and natural sites.

## Introduction

In the face of dramatic biodiversity loss (Butchart *et al.*, 2010; Hoffmann *et al.*, 2010), conservation-based research is being applied to a broad range of disciplines (e.g. reproductive biology, Wildt and Wemmer, 1999; population genetics, Holderegger and Di Giulio, 2010; thermal ecology, Monasterio *et al.*, 2013) and typically includes topics that extend beyond those simply addressing the most obvious threats to species (e.g. habitat destruction, over-harvesting, emerging diseases, invasive species; Wilcove *et al.*, 1998). Road ecology typically focuses on the impacts of the direct threats of road mortality and habitat and population fragmentation on populations (Shepard *et al.*, 2008; Clark *et al.*, 2010). Although these threats are both prevalent and relevant, they are unlikely to be the only negative effects posed by roads to local wildlife populations. If we are to develop a complete understanding of threats to populations posed by roads, we must also look beyond the direct impacts of roads themselves. One such avenue of investigation resides in endocrinology, particularly in promoting the understanding of anthropogenically associated physiological stress (Newcomb Homan *et al.*, 2003; Partecke *et al.*, 2006; Van Meter *et al.*, 2009). Although several studies have examined road-related stress in birds (Crino *et al.*, 2011; Morgan *et al.*, 2012), reptiles, a group whose declines across North America are often associated with roads (23% in the USA, Wilcove *et al.*, 1998; and 41% in Canada, Venter *et al.*, 2006), remain absent from such investigation. Several aspects of physiological stress have been examined in reptiles. For example, stress levels have been examined in relationship to reproductive cycle (lizards, Girling and Cree, 1995; snakes, Graham, 2006), body condition (sea turtles; Jessop *et al.*, 2004), capture/handling (sea turtles, Jessop *et al.*, 2002; snakes, Bailey *et al.*, 2009), invasive species (lizards, Trompeter and Langkilde, 2011) and translocation (tortoises, Drake *et al.*, 2012; snakes, Holding *et al.*, 2014). However, the literature on the effect of urban environments on stress levels is scarce, with the exception of a few seminal studies on desert lizards examining physiological stress levels across a gradient of urbanization (French *et al.*, 2008; Lucas and French, 2012). Roads present a host of potential stressors in the forms of vehicle encounters, as well as sound, light and chemical pollution (Longcore and Rich, 2004), yet the impacts these potential stressors have on the physiological state of reptiles remain unknown.

Physiological stress is the result of an organism's response to negative stimuli through biochemical shifts away from homeostasis in preparation for the physical requirements associated with the stressor (e.g. priming the muscles during a fight-or-flight response; McCarty, 2000; Romero, 2004). Following a period of acute stress, the organism returns to homeostasis through secondary biochemical shifts (Romero, 2004), and in normal conditions, such stress would result in short-term reallocation of energy away from long-term physiological functions (e.g. reproduction, growth, immune function). Alternatively, chronic stress may result in long-term physiological functions becoming disrupted or inhibited (Cabezas *et al.*, 2007; Cyr and Romero, 2007). From a conservation standpoint, understanding the relationship between anthropogenic stressors and chronic stress, as well as the associated implications for population health, is a crucial area of research that will help in determining whether there are indirect threats from anthropogenic structures to populations in decline (Busch and Hayward, 2009).

Levels of glucocorticoids (GCs) are one of the most prominent measurable results of physiological stress. These biochemical products are produced during activation of the hypothalamic–pituitary–adrenal axis (Romero, 2004; French *et al.*, 2008). After encountering a stressor, the release of GCs occurs within a period of minutes to hours, depending on the individual and species (Sapolsky *et al.*, 2000; Moore and Jessop, 2003). The functions of GCs within an organism are wide ranging and even disputed (Sapolsky *et al.*, 2000); however, within the present study we use measurements of GCs to indicate the overall level of stress. In research on reptile stress, the major GC examined is corticosterone (CORT; Sandor, 1972), which is circulated through the bloodstream (Sapolsky *et al.*, 2000). Elevated levels of CORT have been correlated with increased muscular activity within both acute (e.g. during a fight-or-flight response) and cyclic events (e.g. breeding seasons and ovipositing/birthing) for a wide variety of reptiles (Moore and Jessop, 2003). Chronically elevated levels of CORT have been seen to decrease reproductive success and growth (Morici *et al.*, 1997; Moore and Jessop, 2003), underscoring the importance of understanding stress biology for reptile conservation.

Traditionally, sampling CORT in reptiles has relied on blood plasma (French *et al.*, 2008) or faecal sampling (Kalliokoski *et al.*, 2012). Recently, methods have been developed to quantify levels of CORT in keratinized reptile tissues (i.e. snake sheds; Berkvens *et al.*, 2013), and we examined whether CORT could be recovered from the keratinized claw tissue of turtles. As claws grow, CORT in the bloodstream passively diffuses from capillaries within the claw and is deposited into the tissue matrix, incorporating itself within the keratin (Warnock *et al.*, 2010). As CORT is deposited and stored in claw tissue, samples do not demonstrate the same short-term fluctuations as seen in blood plasma (minutes to hours; Romero and Reed, 2005) or faeces (days; Berkvens *et al.*, 2013). Thus, CORT levels recovered from non-vascularized claw samples (i.e. the portion of the claw that has grown away from the blood vessel) represent a static measure of long-term stress. The claw samples can be homogenized, allowing for a long-term average CORT level to be measured, thus providing information relating to chronic physiological stress, as observed with the use of other keratinized tissues (Berkvens, 2012). Furthermore, sampling keratinized tissue is typically less invasive than collecting blood and does not have the confounding issues of circulating CORT levels that may result from animal handling times and capture methods (Berkvens *et al.*, 2013).

In this context, the objectives of our study were as follows: (i) to develop a non-invasive, simple method to determine chronic physiological stress in turtles effectively; and (ii) to use this method in a pilot study examining whether freshwater turtles living around major roadways are experiencing chronic physiological stress. Ultimately, the purpose of this study was to further our ability to detect whether anthropogenic disturbances alter stress levels and to increase our understanding of chronic stress in the context of global reptile population declines.

## Materials and methods

### Sample collection

The following three study sites were used for sample collection: a road-impacted site (Highway 69, a major traffic corridor in central Ontario, Canada); a control site (Neily Lake, Burwash, Ontario, Canada); and a validation site (Magnetawan First Nation, Ontario, Canada). The road-impacted site was a four-lane highway connecting central and southern Ontario, with an average of 9700 vehicles/day during the turtle's active season (MTO, 2010) and a high level of annual reptile road mortality (Baxter-Gilbert, 2014). The road-impacted section of the highway was a construction site for 4 years prior to testing and experienced live traffic for 1 year prior to testing. The control site was located 2.5 km west of Highway 69, at Neily Lake; a small (∼1.3 km long by 0.2 km wide), S-shaped, eutrophic lake surrounded by forest, with a low-use dirt road running along the north shore. The closest potential anthropogenic stressor to the control site was the adjacent Canadian Department of Defense property; however, this property has no personnel living on site, and the small-arms firing range is active only 2–4 days per month and is located 2 km north of the lake. The validation site is a First Nation community in central Ontario bisected by Highway 69; samples from this site were not road dependent and were collected from individuals found on and off the road. The samples from the validation site were used to test the effectiveness of the method, while samples from the road-impacted and control sites were used to examine the effect of roadways on chronic stress in turtles.

The midland painted turtle (*Chrysemys picta marginata*) was our model organism because of its local abundance in both natural areas and around roads (70.2% of the turtles found along the highway were painted turtles; Baxter-Gilbert, 2014). Living and deceased (after being struck by vehicles) adult turtles were collected at the road-impacted site during three daily driving surveys (along 13 km of highway) and a daily walking survey (along 2 km of highway) each day from 1 May to 31 August 2013 (Baxter-Gilbert, 2014). At the control site, turtles were captured via hoop-traps, basking-traps, incidental encounters and dip-netting from a canoe. At the validation site, turtles were captured using all of the capture methods mentioned above.

Claw tips of both hindfeet (eight individual claws) were trimmed from captured turtles using scissor nail-trimmers (Resco, Walled Lake, MI, USA). Care was taken to remove only the first 1–4 mm of claw (depending on wear), which prevented contamination of the sample with blood from the the vessel that runs through the centre of the claw. Claw trimmings were then stored in labelled 20 ml scintillation vials (Fisherbrand, Loughborough, Leicestershire, UK) and stored at room temperature until processing (within 4 months of collection). Following claw sampling, turtles were sexed (juvenile or adult male or female) and weighed using a spring scale (100–2500 g model; Pesola, Barr, Switzerland). Maximal carapace length was measured with callipers (15 cm, Scherr-Tumico, China; 40 cm, Haglof Inc., Langsele, Sweden). Live turtles were individually marked in their marginal scutes using a tapered file (Mastercraft, Toronto, ON, Canada) and a notch code system (Cagle, 1939) to prevent resampling and released at their capture site within 8 h. A total of 15 turtles (four females and 11 males) were sampled at the road-adjacent site, 15 turtles (one female and 14 males) were sampled at the control site, and 24 turtles (eight females, 15 males and one adult of unknown sex) were sampled at the validation site. All field work involving animals adhered to the guidelines of the Canadian Council on Animal Care and an approved Laurentian University Animal Care Committee protocol (AUP# 2013-03-01).

### Hormone extraction

The claw samples used for the validation study were measured with callipers (Scienceware, Pequannock, NJ, USA) and ranged in length from 1.0 to 4.5 mm, with a mean of 2.6 ± 0.1 mm (*n* = 168). Samples were washed and crushed using modifications of methods described previously by Tegethoff *et al.* (2011) and Levitt (1966), respectively. In brief, claws were washed once with 1 ml distilled water and then twice with 1 ml 100% methanol by vortexing for 10 s. Samples were air dried, transferred to 2.0 ml cryovials (Corning Inc., Corning, NY, USA) and placed at −196°C for a minimum of 10 min in a liquid nitrogen dry shipper (Taylor-Wharton, Theodore, AL, USA). Frozen samples were placed in a steel cylinder and given several hard blows with a steel pestle to homogenize the claw sections. The crushed claw pieces were weighed using a Mettler Toledo balance (model AB54-S; ±0.0001 g; Mettler Toledo International, Inc., Columbus, OH, USA) and transferred to 7 ml glass scintillation vials (VWR, Mississauga, ON, Canada). Corticosterone was extracted from the samples in 100% methanol using a ratio of 0.005 g/ml by agitating for 24 h on an orbital shaker (Montreal Biotech Inc., Kirkland, PQ, Canada) at 200 rpm. Samples were then centrifuged at 2300 g for 10 min, and the extract was pipetted off into a new vial. The extract was dried in a fume hood and reconstituted in 150 µl enzyme immunoassay buffer solution (0.1 mm sodium phosphate buffer, pH 7.0, containing 9 g of NaCl and 1 g of bovine serum albumin per litre), resulting in a 1.13- to 16.53-fold concentration. Reconstituted samples were sonicated for 20 s in an Elmasonic waterbath (Elma GmbH & Co. KG, Singen, BW, Germany) and then loaded and incubated on microtitre plates as described by Terwissen *et al.* (2013), before analysis.

Claw CORT values were quantified using modifications of an enzyme immunoassay described previously (Metrione and Harder, 2011; Watson *et al.*, 2013). Antisera were diluted as follows: goat anti-rabbit IgG (GARG) polyclonal antibody (Sigma-Aldrich, Mississauga, ON, Canada), 0.25 µg/well; and CORT polyclonal antibody (CJM006; C. Munro, University of California, Davis, CA, USA), 1:200 000. The cross-reactivities of the antisera have been described previously (GARG and CORT; Metrione and Harder, 2011; Watson *et al.*, 2013). Corticosterone–horseradish peroxidase conjugate (C. Munro, University of California, Davis, CA, USA) was diluted 1:1 000 000. Standard solutions used were created with synthetic CORT (Steraloids Q1550; 39–10 000 pg/ml). The control consisted of a laboratory stock of pooled fecal extracts obtained from spotted-necked otters (*Hydrictis maculicollis*) that was run at 65% binding.

### Enzyme immunoassay validation study

#### Parallelism

Parallel displacements involve examining the relationship between a set of predicted values and test samples, and measuring the variance between them. The standard curve (created from synthetic CORT stock) and a serial dilution (created from turtle claw extract) were used to detect immunological similarities between standard and sample hormones. A pooled sample of claw extracts was concentrated and serially diluted 2-fold from 1:65 to 1:4.1 concentrations in enzyme immunoassay buffer and run alongside the standard curve. Linear regression analysis was used to determine whether there was a significant relationship in the percentage of antibody bound between the standard curve and serial dilutions of the sample extracts.

#### Precision

Intra- and inter-assay coefficients of variation (CVs) were calculated to determine precision and repeatability. To control for intra-assay CV, only values from duplicates with CV < 10% were used. Intra-assay CVs were further evaluated using a pooled extract at 50% binding loaded in different spots on the plate, and this method of evaluation was repeated on three separate plates. Inter-assay CVs were evaluated using a faecal extract control (65% binding; as noted above) loaded in duplicate on each plate.

#### Accuracy

Recovery of a known amount of hormone was calculated to examine possible interference of components within the extract with antibody binding. A pooled sample of claw extract was concentrated to 11-fold concentration. The claw extract was then reconstituted and aliquoted to a volume of 75 µl. Subsequently, a volume of 75 µl of increasing concentrations of CORT standard was then added to the claw extract in the range used for the standard curve. The concentrated pool was assayed alone to determine endogenous hormone levels. The recovery (expressed as a percentage) was calculated using the following formula: amount observed/amount expected × 100, where the observed amount was the value obtained in the spiked sample and the expected amount was the calculated amount of standard hormone added plus the amount of endogenous hormone in the unspiked sample. Linear regression analysis was used to determine whether there was a significant relationship between the hormone added and hormone recovered to assess assay accuracy.

### Pilot field study

Differences in CORT levels extracted from claws were examined using an analysis of variance test (ANOVA) for the fixed effects of site (road impacted, *n* = 15; control, *n* = 15) and sex (male, *n* = 25; female, *n* = 5). A linear regression was used to determine the relationship between CORT levels and body condition (*n* = 18), with sex and location included as covariates (Jessop *et al.*, 2004). Body condition was quantified using the residuals from a regression between turtle mass and maximal carapace length (Schulte-Hostedde *et al.*, 2005; Rasmussen and Litzgus, 2010). The residuals were evenly distributed across the range of carapace lengths in the study; thus, we could assume that the relationship was linear (Schulte-Hostedde *et al.*, 2005). All statistical tests for this study were conducted in R statistical software (version 2.15.0, R Development Core Team 2012). All summary data are reported as means ± SEM. All statistical analyses tested for interactions, but if the interactions were not significant only main effects were reported. A significance level of α = 0.05 was used for all statistical tests.

## Results

### Enzyme immunoassay validation study

**Figure 1:**
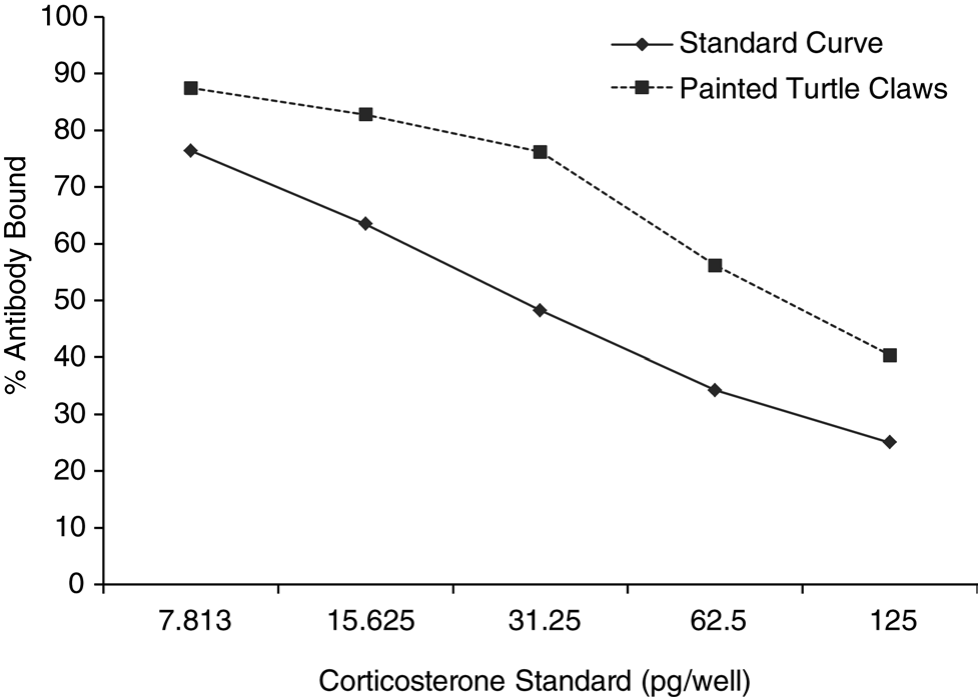
Parallelism between standard curve and serial dilutions of sample corticosterone (CORT) extract. A significant relationship was found in the amounts of antibody bound to CORT between the painted turtle samples and the standard solutions created from synthetic stock (*r*^2^ = 0.952, *P* < 0.01).

**Figure 2:**
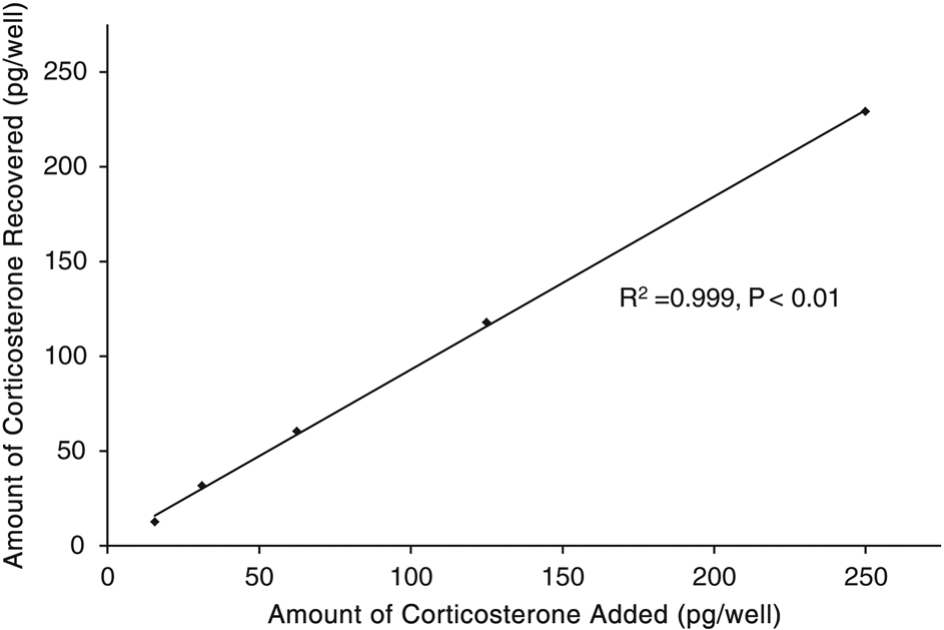
Recovery of exogenous CORT from turtle claw extracts, demonstrating a significant relationship between the amounts of CORT recovered from samples with varying amounts of spiked CORT (*P* < 0.01).

Serial dilutions of pooled turtle claw extract were significantly related to the CORT standard curve (*r*^2^ = 0.95, *P* < 0.01), indicating parallel displacement (Fig. 1). The recovery of known concentrations of CORT in turtle claw extracts was 92.8 ± 3.5%. The measured hormone concentrations in the spiked samples were correlated with the expected concentrations (*r*^2^ = 0.99, *P* < 0.01; Fig. 2). Intra-assay CV (variation within plates) was 5.6% at 50% binding, while inter-assay CV (variation between plates) was 3.3% at 65% binding.

### Pilot field study

**Figure 3:**
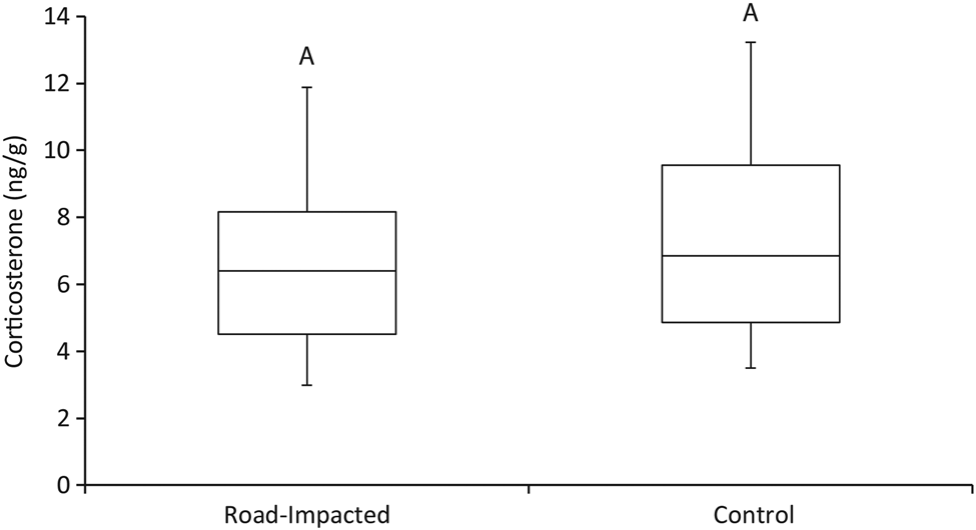
Average amount of CORT recovered from claw samples collected from turtles living alongside roads (road impacted; *n* = 15) and at a more natural site (control, *n* = 15). Common letters indicate no significant difference.

**Figure 4:**
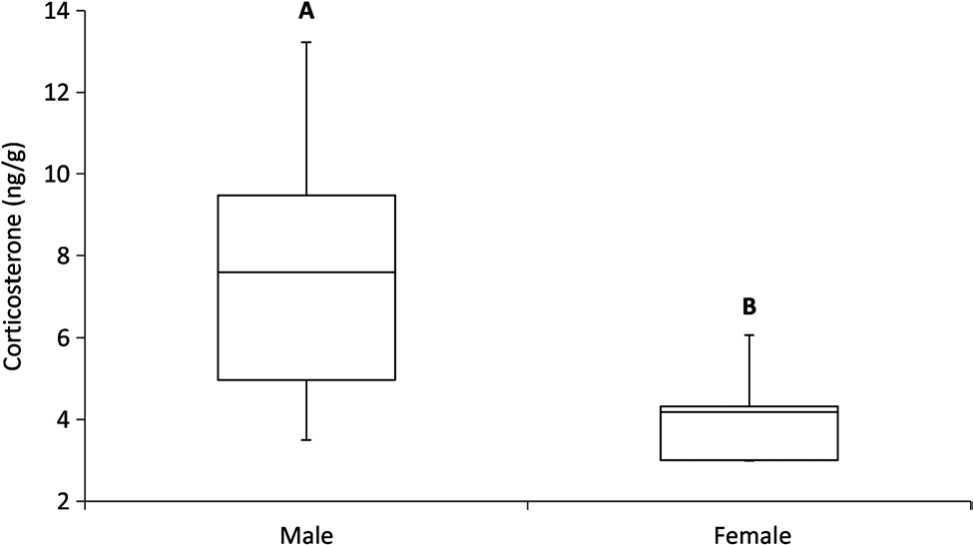
Average amount of CORT collected from claw samples for female (*n* = 5) and male painted turtles (*n* = 25). Unique letters represent a significant difference.

There was no difference in the amount of CORT between road-impacted and control sites (*F*_1,27_ = 0.30, *P* = 0.59). The amount of CORT detected in claws from turtles (presented as nanograms of CORT per gram of claw) was 6.46 ± 0.69 and 7.69 ± 0.81 ng/g at the road-impacted site and control site, respectively (Fig. 3). Females had significantly less CORT in their claws than males (*F*_1,27_ = 6.08, *P* = 0.02; Fig. 4). On average, females had 4.11 ± 0.56 ng/g of CORT in their claws, while males had 7.66 ± 0.56 ng/g. Finally, no significant relationship was found between the residual index of body condition and CORT levels (*F*_3,14_ = 0.45, *P* = 0.72); the data were widely distributed and exhibited no directional trend.

## Discussion

### Enzyme immunoassay validation study

The validation study demonstrated the ability to extract reliable levels of CORT from turtle claws. This novel technique can be used simply and non-invasively to collect samples that reflect long-term CORT levels for the examination of chronic physiological stress. Interestingly, the average recovered amount of baseline CORT from claw samples (7.07 ng/g) fell within a range comparable with the baseline amounts of CORT recovered from blood samples for other groups of reptiles [between 2 and 12 ng/ml seen in green sea turtles (Cheloniamydas), garter snakes (*Thamnophis sirtalis*) and marine iguanas (*Amblyrhynchus cristatus*); Moore and Jessop, 2003]. However, our values were much higher than those reported by Selman *et al.* (2012), who found mean baseline CORT levels of 0.04 ng/ml for yellow-blotched sawback turtles (*Graptemys flavimaculata*); those authors suggested that circulating CORT levels might be much lower in freshwater turtles than in other reptiles.

Recently, the use of keratinized structures (e.g. hairs, feathers and snakes shed skins) has provided valuable information regarding long-term physiological stress in a number of mammals, birds and snakes (Berkvens, 2012). The use of claw samples to measure CORT will expand the number of organisms for which keratinized tissues can be used to evaluate chronic stress; however, further validation of this technique is required. A comparison of blood and faecal CORT concentrations with claw levels is necessary to determine a relationship between these measurements, as shown by Berkvens *et al.* (2013) in snake faeces and shed skins. Furthermore, although logistical constraints prohibited the measurement of claw growth rates in the populations we sampled, this information is necessary to determine the period of time represented by the claw sample and thus help to disentangle chronic vs. acute stress. In our study, the time period represented by the claw sample can be estimated only coarsely (i.e. we assume that the 2–4 mm of claw we used represents a period ranging from months to a year). However, we believe that our coarse estimation of time was sufficient for our objectives to validate this method of sampling CORT and to conduct a pilot study to compare turtles living in similar environmental conditions differentiated only by the presence of a major roadway. Ultimately, the use of keratinized tissue will provide a new means for wildlife managers and conservation biologists to study stress in clawed reptiles using a minimally invasive and easily executed sampling technique, and examinination of stress is an important proxy for population health.

### Pilot field study

In examining average baseline CORT levels extracted from claw samples, our pilot study is the first to test whether roads act as a chronic stressor for freshwater turtles. Although only a small number of individual turtles were sampled, we achieved interesting preliminary results. Our hypothesis was rejected, because we did not find a difference in CORT levels between the road-impacted and control sites. Two possible explanations for this result are as follows: (i) there was an unknown additional stressor at the control site; or (ii) painted turtles may not have elevated stress levels due to highways. The control site is located adjacent to a small, low-use dirt road and a limited-use military training ground, providing occasional disturbance. Nevertheless, the control site did not experience anywhere near the constant levels of disturbance or the volume of noise pollution present around the highway at the road-impacted site. Although the possibility of unknown additional stressors exists, we feel that the second explanation (i.e. painted turtles do not suffer from elevated stress levels due to highways) is more likely. Painted turtles are common in anthropogenic environments, are often seen crossing roads (Baxter-Gilbert, 2014) and are the only turtle species in Ontario, Canada not listed as a ‘species at risk’ (COSEWIC, 2011). Within a conservation framework, our pilot study is a ‘proof of concept’ of our ability to examine reptile stress easily. However, our investigation was restricted to one road site and one control site, limiting the inferences that can be made from this study alone. Further research is required, using multiple replicates from numerous sites and across varying levels of anthropogenic disturbance, before a conclusive statement can be made regarding the potential for roads and traffic to alter stress levels in painted turtles. Additionally, more research is required on rare and threatened species in order to determine whether they present a more severe response to road-altered environments than more common species. Only through a better understanding of the threats, including indirect ones, will we be able to ensure the protection and recovery of imperilled species.

In addition to the comparison of CORT levels made between the two study sites used to test our hypothesis, we also examined the relationship between body condition and baseline stress levels, which is relatively under-studied in freshwater turtles. Body condition was related to baseline levels of CORT in green sea turtles but not in hawksbill sea turtles (*Eretmochelys imbricate*; Jessop *et al.*, 2004). Here, we did not observe a relationship between our long-term measure of stress and body condition for painted turtles. A potential explanation of our results may be that shifts in baseline CORT levels related to body condition occur only once an individual has reached a certain threshold (i.e. malnourished or significantly overweight), and that the individuals we measured were simply within the normal scope for the species. However, further research is required to test the validity of this explanation. Furthermore, one could consider an alternative to examining the relationship between body condition and stress by investigating the response of immune function to stress. Berger *et al.* (2005) examined immunocompetence of marine iguanas at varying levels of induced stress (both chemical and physical) and determined that immune function was significantly decreased during periods of elevated CORT levels. Future studies should examine whether turtles also exhibit a relationship between immune function and CORT levels, which would determine whether immune function may represent a better proxy than body condition for examining the effects of elevated CORT levels.

In our study, we also observed significantly lower long-term CORT levels in females than in males, albeit we had a relatively small female sample size (*n* = 5). Increased levels of CORT in males may be attributed to reproductively beneficial traits, as seen in marine iguanas (e.g. orientation, territoriality and courtship behaviour; Berger *et al.*, 2005); however, the relationship between CORT and similar traits in painted turtles remains unknown. Conversely, it is possible that females may be exhibiting reduced CORT levels, as seen with post-nesting season green sea turtles (Hamann *et al.*, 2002), because most of our females were captured after the nesting season. Within some species of freshwater turtles (e.g. yellow-blotched sawback turtles; Selman *et al.*, 2012), CORT levels vary for both males and females, and the difference depends on seasonal energy reallocation (e.g. spring or autumn breeding, nesting, overwintering migrations); however, even though seasonally acute shifts were documented in yellow-blotched sawback turtles, overall CORT levels were not significantly different over the active season for either sex (Selman *et al.*, 2012). Our method of sampling CORT would not capture short-term seasonal fluctuations, but instead reflects sex-specific differences in baseline CORT averaged over the long term. Cash *et al.* (1997) noted no difference in baseline CORT between the sexes in red-eared sliders (*Trachemys scripta*), suggesting that perhaps our findings may have simply been a result of the low female sample size. In fact, differences between the sexes in baseline levels of CORT are inconsistent within reptiles. A difference in sex-specific baseline CORT levels has been observed for some reptiles [watersnakes (*Nerodia sipedon*), Sykes and Klukowski, 2009; common wall lizards (*Podarcis muralis*), Galeotti *et al.*, 2010] but not for others [freshwater crocodiles (*Crocodylus johnstoni*), Jessop *et al.*, 2003; hawksbill turtles, Jessop *et al.*, 2004; tree lizards (*Urosaurus ornatus*), French *et al.*, 2008]. To our knowledge, documentation of stress levels in freshwater turtles is represented by only a handful of studies (Cash *et al.*, 1997; Selman *et al.*, 2012). Furthermore, the literature on stress in freshwater turtles has yet to address chronic stress and the potential for chronic stress to have negative population effects. Turtles are the most imperilled group of reptiles (Gibbons *et al.*, 2000; Böhm *et al.*, 2013); thus, it is imperative that we increase our understanding of the relationships among stress, body condition, individual fitness and overall population health so that we can better conserve this unique taxon (Wikelski and Romero, 2003; Bonier *et al.*, 2009; Selman *et al.*, 2012).

### Conclusion

In the conservation of imperilled species, it is critically important to understand the physiological responses of individuals to anthropogenic threats to their populations, such as habitat degradation, urbanization and climate change (Newcomb Homan *et al.*, 2003; Partecke *et al.*, 2006; Van Meter *et al.*, 2009; Satterthwaite *et al.*, 2012). By understanding the physiological effects (e.g. chronic stress) caused by human disturbance, conservation actions can be directed towards understanding the indirect threats, which have until recently remained unstudied (Ellis *et al.*, 2012). The assessment of such indirect effects has broad implications for conservation biology; for instance, when examining the effects of roads on imperilled species, it may be important to determine whether population declines around roads are due to direct threats (such as high road mortality; Aresco, 2005), indirect threats (such as chronic physiological stress) or a synergistic combination of both.

While the potential for anthropogenic disturbance to cause chronic stress in organisms remains understudied (Johnstone *et al.*, 2012), we have evaluated a novel, simple and non-invasive method to examine levels of long-term CORT in turtles. This novel method provides a unique opportunity to detect whether specific populations experience chronic stress as a result of human disturbance. Ultimately, conservation biologists need to identify and understand the presence and diversity of threats to an imperilled species before proper and effective mitigation can be developed and implemented.

## References

[COU036C1] Aresco MJ (2005) Mitigation measures to reduce highway mortality of turtles and other herpetofauna at a north Florida lake. J Wildlife Manage 69: 549–560.

[COU036C2] Bailey FC Cobb VA Rainwater TR Worrall T Klukowski M (2009) Adrenocortical effects of human encounters on free-ranging Cottonmouths (*Agkistrodon piscivorus*). J Herpetol 43: 260–266.

[COU036C3] Baxter-Gilbert JH (2014) The long road ahead: understanding road-related threats to reptiles and testing if current mitigation measures are effective at minimizing impacts. Master's thesis. Laurentian University, Sudbury, Ontario, Canada.

[COU036C4] Berger S Martin LB II Wikelski M Romero LM Kalko EK Vitousek MN Rödl T (2005) Corticosterone suppresses immune activity in territorial Galapagos marine iguanas during reproduction. Horm Behav 47: 419–429. 15777807 10.1016/j.yhbeh.2004.11.011

[COU036C5] Berkvens CN (2012) Keratin glucocorticoid analysis by enzyme immunoassay in mammals, birds and reptiles. PhD thesis. University of Guelph, Guelph, Ontario, Canada.

[COU036C6] Berkvens CN Hyatt C Gilman C Pearl DL Barker IK Mastromonaco GF (2013) Validation of a shed skin corticosterone enzyme immunoassay in the African House Snake (*Lamprophis fuliginosus*) and its evaluation in the Eastern Massasauga Rattlesnake (*Sistrurus catenatus catenatus*). Gen Comp Endocrinol 194: 1–9. 23994033 10.1016/j.ygcen.2013.08.011

[COU036C7] Böhm M Collen B Baillie JEM Bowles P Chanson J Cox N Hammerson G Hoffmann M Livingstone SR Ram M (2013) The conservation status of the world's reptiles. Biol Conserv 157: 372–385.

[COU036C8] Bonier F Martin PR Moore IT Wingfield JC (2009) Do baseline glucocorticoids predict fitness? Trends Ecol Evol 24: 634–642. 19679371 10.1016/j.tree.2009.04.013

[COU036C9] Busch DS Hayward LS (2009) Stress in a conservation context: a discussion of glucocorticoid actions and how levels change with conservation-relevant variables. Biol Conserv 142: 2844–2853.

[COU036C10] Butchart SHM Walpole M Collen B van Strien A Scharlemann JPW Almond REA Baillie JEM Bomhard B Brown C Bruno J (2010) Global biodiversity: indicators of recent declines. Science 328: 1164–1168. 20430971 10.1126/science.1187512

[COU036C11] Cabezas S Blas J Marchant TA Moreno S (2007) Physiological stress levels predict survival probabilities in wild rabbits. Horm Behav 51: 313–320. 17258747 10.1016/j.yhbeh.2006.11.004

[COU036C12] Cagle FR (1939) A system for marking turtles for future identification. Copeia 1939: 170–173.

[COU036C13] Cash WB Holberton RL Knight SS (1997) Corticosterone secretion in response to capture and handling in free-living red-eared slider turtles. Gen Comp Endocrinol 108: 427–433. 9405119 10.1006/gcen.1997.6999

[COU036C14] Clark RW Brown WS Stechert R Zamudio KR (2010) Roads, interrupted dispersal, and genetic diversity in timber rattlesnakes. Conserv Biol 24: 1059–1069. 20151984 10.1111/j.1523-1739.2009.01439.x

[COU036C15] COSEWIC (2011) Canadian Wildlife Species at Risk. Committee on the Status of Endangered Wildlife in Canada 2011 Report. Gatineau, Quebec, Canada.

[COU036C16] Crino OL Van Oorschot BK Johnson EE Malisch JL Breuner CW (2011) Proximity to a high traffic road: glucocorticoid and life history consequences for nestling white-crowned sparrows. Gen Comp Endocrinol 173: 323–332. 21712039 10.1016/j.ygcen.2011.06.001

[COU036C17] Cyr NE Romero LM (2007) Chronic stress in free-living European starlings reduces corticosterone concentrations and reproductive success. Gen Comp Endocrinol 151: 82–89. 17280663 10.1016/j.ygcen.2006.12.003

[COU036C18] Drake KK Nussear KE Esque TC Barber AM Vittum KM Medica PA Tracy CA Hunter KW (2012) Does translocation influence physiological stress in the desert tortoise? Anim Conserv 15: 560–570.

[COU036C19] Ellis RD McWhorter TJ Maron M (2012) Integrating landscape ecology and conservation physiology. Landscape Ecol 27: 1–12.

[COU036C20] French SS Fokidis HB Moore MC (2008) Variation in stress and innate immunity in the tree lizard (*Urosaurus ornatus*) across an urban–rural gradient. J Comp Physiol B 178: 997–1005. 18594834 10.1007/s00360-008-0290-8 PMC2774757

[COU036C21] Galeotti P Pellitteri-Rosa D Sacchi R Gentilli A Pupin F Rubolini D Fasola M (2010) Sex-, morph- and size-specific susceptibility to stress measured by haematological variables in captive common wall lizard *Podarcis muralis*. Comp Biochem Physiol A Mol Integr Physiol 157: 354–363. 20713170 10.1016/j.cbpa.2010.08.005

[COU036C22] Gibbons JW Scott DE Ryan TJ Buhlmann KA Tuberville TD Metts BS Greene JL Mills T Leiden Y Poppy S (2000) The global decline of reptiles, déjà vu amphibians. BioScience 50: 653–666.

[COU036C23] Girling JE Cree A (1995) Plasma corticosterone levels are not significantly related to reproductive stage in female common geckons (*Hoplodactylus maculatus*). Gen Comp Endocrinol 100: 273–281. 8775054 10.1006/gcen.1995.1158

[COU036C24] Graham SP (2006) An integrative analysis of reproduction and stress in free-living male cottonmouths, Agkistrodon piscivorus. MSc. thesis. Center for Behavioral Neuroscience, Georgia State University, GA, USA.

[COU036C25] Hamann M Jessop T Limpus C Whittier J (2002) Interactions among endocrinology, seasonal reproductive cycles and the nesting biology of the female green sea turtle. Mar Biol 140: 823–830.

[COU036C26] Hoffmann M Hilton-Taylor C Angulo A Böhm M Brooks TM Butchart SHM Carpenter KE Chanson J Collen B Cox NA (2010) The impact of conservation on the status of the world's vertebrates. Science 330: 1503–1509. 20978281 10.1126/science.1194442

[COU036C27] Holderegger R Di Giulio M (2010) The genetic effects of roads: a review of empirical evidence. Basic Appl Ecol 11: 522–531.

[COU036C28] Holding ML Frazier JA Dorr SW Henningsen SN Moore IT Taylor EN (2014) Physiological and behavioral effects of repeated handling and short-distance translocations on free-ranging northern Pacific rattlesnakes (*Crotalus oreganus oreganus*). J Herpetol 48: 233–239.

[COU036C29] Jessop TS Knapp R Whittier JM Limpus CJ (2002) Dynamic endocrine responses to stress: evidence for energetic constraints and status dependence in breeding male green turtles. Gen Comp Endocrinol 126: 59–67. 11944967 10.1006/gcen.2001.7769

[COU036C30] Jessop TS Tucker AD Limpus CJ Whittier JM (2003) Interactions between ecology, demography, capture stress, and profiles of corticosterone and glucose in a free-living population of Australian freshwater crocodiles. Gen Comp Endocrinol 132: 161–170. 12765656 10.1016/s0016-6480(03)00078-9

[COU036C31] Jessop TS Sumner JM Limpus CJ Whittier JM (2004) Interplay between plasma hormone profiles, sex and body condition in immature hawksbill turtles (*Eretmochelys imbricata*) subjected to a capture stress protocol. Comp Biochem Physiol A Mol Integr Physiol 137: 197–204. 14720605 10.1016/j.cbpb.2003.09.029

[COU036C32] Johnstone CP Lill A Reina RD (2012) Does habitat fragmentation cause stress in the agile antechinus? A haematological approach. J Comp Physiol B 182: 139–155. 21710385 10.1007/s00360-011-0598-7

[COU036C33] Kalliokoski O Timm JA Ibsen IB Hau J Frederiksen AMB Bertelsen MF (2012) Fecal glucocorticoid response to environmental stressors in green iguanas (*Iguana iguana*). Gen Comp Endocrinol 177: 93–97. 22414390 10.1016/j.ygcen.2012.02.017

[COU036C34] Levitt JI (1966) Creatinine concentration of human fingernail and toenail clippings. Ann Intern Med 64: 312–327. 5902278 10.7326/0003-4819-64-2-312

[COU036C35] Longcore T Rich C (2004) Ecological light pollution. Front Ecol Environ 2: 191–198.

[COU036C36] Lucas LD French SS (2012) Stress-induced tradeoffs in a free-living lizard across a variable landscape: consequences for individuals and populations. PLoS ONE 7: e49895. 10.1371/journal.pone.0049895 PMC3502225 23185478

[COU036C37] McCarty R (2000) Flight or fight response. In Fink G, ed, Encyclopedia of Stress, Vol 2 Academic Press, London, UK, pp 143–145.

[COU036C38] Metrione LC Harder JC (2011) Fecal corticosterone concentrations and reproductive success in captive female southern white rhinoceros. Gen Comp Endocrinol 171: 283–292. 21354160 10.1016/j.ygcen.2011.02.010

[COU036C39] Monasterio C Shoo LP Salvador A Iraeta P Díaz JA (2013) High temperature constrains reproductive success in a temperate lizard: implications for distribution range limits and the impacts of climate change. J Zool 291: 136–145.

[COU036C40] Moore IT Jessop TS (2003) Stress, reproduction, and adrenocortical modulation in amphibians and reptiles. Horm Behav 43: 39–47. 12614633 10.1016/s0018-506x(02)00038-7

[COU036C41] Morgan GM Wilcoxen TE Rensel MA Schoech SJ (2012) Are roads and traffic sources of physiological stress for the Florida scrub-jay? Wildlife Res 39: 301–310.

[COU036C42] Morici LA Elsey RM Lance VA (1997) Effects of long–term corticosterone implants on growth and immune function in juvenile alligators, *Alligator mississippiensis*. J Exp Zool 279: 156–162. 9293640

[COU036C43] MTO (2010) Provincial Highway Traffic Volume 2010. Ontario Ministry of Transportation, Highway Standards Branch. Queen's Printer for Ontario, Toronto, Ontario, Canada.

[COU036C44] Newcomb Homan R Regosin JV Rodrigues DM Reed JM Windmiller BS Romero LM (2003) Impacts of varying habitat quality on the physiological stress of spotted salamanders (*Ambystoma maculatum*). Anim Conserv 6: 11–18.

[COU036C45] Partecke J Schwabl I Gwinner E (2006) Stress and the city: urbanization and its effects on the stress physiology in European blackbirds. Ecology 87: 1945–1952. 16937632 10.1890/0012-9658(2006)87[1945:satcua]2.0.co;2

[COU036C46] Rasmussen ML Litzgus JL (2010) Patterns of maternal investment in spotted turtles (*Clemmys guttata*): implications of trade-offs, scales of analyses, and incubation substrates. Ecoscience 17: 47–58.

[COU036C47] Romero LM (2004) Physiological stress in ecology: lessons from biomedical research. Trends Ecol Evol 19: 249–255. 16701264 10.1016/j.tree.2004.03.008

[COU036C48] Romero LM Reed JM (2005) Collecting baseline corticosterone samples in the field: is under 3 min good enough? Comp Biochem Physiol A Mol Integr Physiol 140: 73–79. 15664315 10.1016/j.cbpb.2004.11.004

[COU036C49] Sandor T (1972) Corticosteroids in amphibia, reptilia and aves. In DR Idler, ed, Steroids in Nonmammalian Vertebrates. Academic Press, New York, USA, pp 253–323.

[COU036C50] Sapolsky RM Romero LM Munck AU (2000) How do glucocorticoids influence stress responses? Integrating permissive, suppressive, stimulatory, and preparative actions. Endocr Rev 21: 55–89. 10696570 10.1210/edrv.21.1.0389

[COU036C51] Satterthwaite WH Kitaysky AS Mangel M (2012) Linking climate variability, productivity and stress to demography in a long-lived seabird. Mar Ecol Prog Ser 454: 221–235.

[COU036C52] Schulte-Hostedde AI Zinner B Millar JS Hickling GJ (2005) Restitution of mass-size residuals: validating body condition indices. Ecology 86: 155–156.

[COU036C53] Selman W Jawor JM Qualls CP (2012) Seasonal variation of corticosterone levels in *Graptemys flavimaculata*, an imperiled freshwater turtle. Copeia 2012: 698–705.

[COU036C54] Shepard DB Kuhns AR Dreslik MJ Phillips CA (2008) Roads as barriers to animal movement in fragmented landscapes. Anim Conserv 11: 288–296.

[COU036C55] Sykes KL Klukowski M (2009) Effects of acute temperature change, confinement and housing on plasma corticosterone in water snakes, *Nerodia sipedon* (Colubridae: Natricinae). J Exp Zool A Ecol Genet Physiol 311: 172–181. 19051318 10.1002/jez.515

[COU036C56] Tegethoff M Raul JS Jamey C Ben Khelil M Ludes B Meinlschmidt B (2011) Dehydroepiandrosterone in nails of infants: a potential biomarker of intrauterine responses to maternal stress. Biol Psychol 87: 414–420. 21645584 10.1016/j.biopsycho.2011.05.007

[COU036C57] Terwissen CV Mastromonaco GF Murray DL (2013) Influence of adrenocorticotrophin hormone challenge and external factors (age, sex, and body region) on hair cortisol concentration in Canada lynx (*Lynx canadensis*). Gen Comp Endocrinol 194: 162–167. 24080086 10.1016/j.ygcen.2013.09.010

[COU036C58] Trompeter WP Langkilde T (2011) Invader danger: lizards faced with novel predators exhibit an altered behavioral response to stress. Horm Behav 60: 152–158. 21549122 10.1016/j.yhbeh.2011.04.001

[COU036C59] Van Meter PE French JA Dloniak SM Watts HE Kolowski JM Holekamp KE (2009) Fecal glucocorticoids reflect socio-ecological and anthropogenic stressors in the lives of wild spotted hyenas. Horm Behav 55: 329–337. 19056392 10.1016/j.yhbeh.2008.11.001 PMC2987620

[COU036C60] Venter O Brodeur NN Nemiroff L Belland B Dolinsek IJ Grant JW (2006) Threats to endangered species in Canada. BioScience 56: 903–910.

[COU036C61] Warnock F McElwee K Mcisaac S Seim D Macritchie KA Young AH (2010) Measuring cortisol and DHEA in fingernails: a pilot study. Neuropsychiatr Dis Treat 6: 1–7. 20169040 PMC2951060

[COU036C62] Watson R Munro C Edwards KL Norton V Brown JL Walker SL (2013) Development of a versatile enzyme immunoassay for non-invasive assessment of glucocorticoid metabolites in a diversity of taxonomic species. Gen Comp Endocrinol 186: 16–24. 23462197 10.1016/j.ygcen.2013.02.001

[COU036C63] Wikelski M Romero LM (2003) Body size, performance and fitness in Galapagos marine iguanas. Integr Comp Biol 43: 376–386. 21680446 10.1093/icb/43.3.376

[COU036C64] Wilcove DS Rothstein D Dubow J Phillips A Losos E (1998) Quantifying threats to imperiled species in the United States. BioScience 48: 607–615.

[COU036C65] Wildt DE Wemmer C (1999) Sex and wildlife: the role of reproductive science in conservation. Biodivers Conserv 8: 965–976.

